# *Moringa oleifera* Use in Maintaining Oral Health and Its Potential Use in Regenerative Dentistry

**DOI:** 10.1155/2023/8876189

**Published:** 2023-10-17

**Authors:** Nada E. Shafiq, Anas F. Mahdee

**Affiliations:** Restorative and Aesthetic Dentistry Department, College of Dentistry, University of Baghdad, Baghdad, Iraq

## Abstract

Phytomedicine refers to the use of naturally derived products to cure and mitigate human conditions. Natural products have the advantages of causing minimum side effects, being biocompatible, available, and economical, with a wide array of biological activities. Reports have described the use of natural products with antimicrobial and anti-inflammatory properties to treat oral conditions and promote wound healing. *Moringa oleifera*, known as the “drumstick” or “horseradish” tree, is believed to have medicinal properties regarding a range of medical conditions, though there is limited information on its use in oral medicine. This narrative review focuses on the use of *Moringa* extracts in the management of oral conditions, including oral infections, inflammatory conditions, the remineralization of hard tissues, oral wound healing, and tissue regeneration, drawing from both *in vitro* and *in vivo* studies which indicate that the potential of *Moringa* extracts in supporting dentin-pulp regeneration after caries or trauma is worthy of more careful consideration.

## 1. Introduction

Phytomedicine refers to a body of therapeutic knowledge based on natural extracts of plants, animals, fungi, and minerals [1]. Reports are available describing the use of “natural” products in the treatment of gingivitis and periodontitis, promoting periodontal wound healing after traumatic injury or pathologic disease [2], the healing of oral ulcers, pulp and periapical treatment [3], and therapeutic use for oral cancer both *in vitro* and *in vivo* [4–6].

*Moringa oleifera* (*M. oleifera*) is a mineral and vitamin rich, nutritious, and medicinally important tree species, belonging to the family Moringaceae. Common names include drumstick, ben oil, or horseradish tree [7]. Its cultivation is widespread in the Himalayan foothills, with subsequent introduction to south east and west Asia, Arabian lands, east and west Africa, some states of the USA, and South America [8]. *M. oleifera* is considered to be a “super” plant by virtue of its exceptional properties to combat various illnesses in the human body [9].

The phytochemistry of *M. oleifera* reveals different classes of compounds with the potential to confer health benefits. The presence and amount of these compounds vary according to the geographical location of cultivation, soil type, climatic conditions, and sun exposure [10]. Derivatives of this plant may also vary according to extraction methods, especially the use of different solvents including methanol, ethanol, and water [11, 12]. Numerous bioactive compounds can be extracted from this plant: flavonoids, phenolic acids, glucosinolates, saponins, tannins, steroids, alkaloids, and terpenes [13]. The flavonoids present include rutin, quercetin, rhamnetin, kaempferol, apigenin, and myricetin [14], and these compounds have numerous therapeutic effects including anti-inflammatory, antioxidant, antibacterial, and hypoglycemic [15] and help in wound healing and tissue regeneration [16]. In addition, numerous phenolic acids are present in leaves, including caffeic acid, chlorogenic acid, gallic acid, and others [13, 17]. Furthermore, all parts of the *M. oleifera* plant contain glucosinolates, giving the plant its potential cancer chemopreventive [18, 19], hypotensive and antibacterial [20, 21], and potential activities against neurodegenerative diseases [22]. Tannins are proposed to contribute to anticancer, antimicrobial, and antihepatoxic activities [23]. *Moringa* also contains alkaloids with calcium-channel blocking activity used in antihypertensive therapy [24]. Moreover, the plant leaf and seed extracts were reported to be potent natural coagulants in the water purification process [25].

Different traditional and nontraditional methods have been developed for extraction of the active components from *M. oleifera* leaves and seeds [26]. Traditional solvent extraction using ethanol and methanol is effective for production of *M. oleifera* leaf extract with high yield and the highest contents of phenolic compounds and highly antioxidant flavonoids [11, 12]. Other modern nontraditional methods, such as ultrasound [27] and microwave-assisted [28] extraction, have also been employed. These different methods have been reported in the fabrication of various formulations of *M. oleifera* extracts to be used for therapeutic purposes. Examples of such products include the nanomicelle of seed oil to treat cancer [29], herbal nasal gels for the treatment of allergic rhinitis [30], alginate-pectin film dressing containing extracts of *M. oleifera* for wound healing application [31], seed oil cream with anti-inflammatory properties [32], and granules of *Moringa* extract as antiarthritic therapy [33].

Recently, active molecules have been incorporated into dental materials with the objective of simulation of the dentin-pulp complex behavior to promote repair and regeneration and manage the inflammatory process and the deposition of mineralized tissue [34]. Therefore, the purpose of this narrative review is to present a comprehensive summary of the properties of *M. oleifera* extracts which may be beneficial in the regeneration of tissues and specifically in the damaged dental pulp.

## 2. Search Strategy

An electronic search of PubMed and Science Direct databases was undertaken using keywords including combinations of “*Moringa oleifera*” with “dental,” “dentistry,” and “regeneration.” The search of articles published in English was conducted from January 2010 to July 2022. The identified articles were filtered according to their relevance to dental aspects and regeneration, and the bibliographies of relevant articles were manually searched for additional articles of interest. The search was limited to original articles, research papers, case reports, and reviews of the literature. Materials published as letters to the editor, website entries, and social media posts were excluded. The search resulted in collection of 113 initial potential articles, and after excluding duplicates (*n* = 17), the remaining articles were initially filtered by reading their titles, abstracts, and full texts to identify 29 articles that fitted the scope of the review.

## 3. Antimicrobial Activity of *Moringa oleifera* against Oral Pathogens

The antibacterial activity of the methanolic extract of *M. oleifera* inhibited the growth of *Streptococcus mutans*, *Streptococcus salivarius*, *Streptococcus mitis*, *Lactobacillus fermentum*, *Streptococcus anginosus*, *Streptococcus gordonii*, *Lactobacillus acidophilus*, and *Staphylococcus aureus* using the *in vitro* well diffusion method [35]. Elgamily et al. [36] reported that extracts from different parts of *Moringa* (leaves, roots, seeds, and their mixture) showed antibacterial activity against *Staphylococcus aureus* and *Streptococcus mutans* growth, although there was no inhibition of oral fungus *Candida albicans*. In the same study, the incorporation of ethanolic extract from leaves with a passive tooth paste and mouth wash significantly increased their antimicrobial effectiveness. An *in vitro* study performed by Arévalo-Híjar et al. [37] found that methanolic extracts of *M. oleifera* leaves suppressed the growth of *Enterococcus faecalis* with a greater antibacterial effect in comparison to 2% chlorhexidine. These extracts showed minimal cytotoxic effects on cultured Madin–Darby canine kidney (MDCK) cells. This study suggested the possible use of these extracts as antimicrobial agents in root canal therapy. Aqueous and ethyl alcohol extracts from *M. oleifera* leaves were tested *in vitro* for their antimicrobial activity on biofilms from volunteer specimens against *Streptococcus mutans* in comparison to 70% ethanol. Both extracts displayed powerful antimicrobial effects and restrained cariogenic biofilm formation, suggesting that they could be employed as preventive measures for dental caries [38]. Another study by Noushad et al. [39] demonstrated a significant antibacterial effect of aqueous extract of *M. oleifera* seeds against *Enterococcus faecalis* bacterial growth in comparison to 5.25% sodium hypochlorite by using an agar diffusion test.

These reports demonstrated *in vitro* antibacterial activity of *M. oleifera* against caries-related bacteria and inhibition of artificially grown cariogenic biofilms. It is important to note that some of these studies had no conventional controls for comparison and that the methods of evaluation may not ideally represent *in vivo* conditions. Further work is clearly required to optimize the extraction of active ingredients, identify and purify potentially useful compounds, and conduct more rigorous evaluation using test models that reflect *in vivo* conditions. This should be combined with careful investigation of the safety and toxicity of these agents prior to their application.

## 4. Effects of *Moringa* in Oral Inflammatory Conditions

Dos Santos et al. [40] studied natural isothiocyanate from *M. oleifera* and its seven analogue molecules (semisynthetic of derivatives MC-D1, MC-D6, MC-D7, MC-D8, and MC-D9) in a TMJ inflammatory hypernociception model in rats. They found that MC-D7 and MC-D9 were effective in reducing nociception and formalin-induced inflammation, while MC-H was more effective against serotonin-induced hypernociception in comparison to indomethacin as a positive control. In another *in vivo* study of temporomandibular joint pain using male Wistar rats, the semisynthetic derivative MC-H exhibited antinociceptive and anti-inflammatory effects when administered orally. This potential analgesic effect could be peripherally mediated by action of the heme oxygenase-1 (HO-1) pathway, as well as through inhibition of intercellular adhesion molecule levels while centrally by the activation of opioid receptors (*µ* and *δ*) [41].

In a study employing network pharmacology and molecular docking, phenolic compounds derived from *M. oleifera* leaf were investigated for antiperiodontitis effects both in an *in vitro* RAW 264.7 macrophage cell culture and in an *in vivo* ligature-induced periodontitis rat model. *Moringa* leaf extract achieved antiperiodontitis activity by regulating the p38*α*/MAPK14-OPG/RANKL pathway. The extract also decreased serum proinflammatory cytokines and increased anti-inflammatory cytokines and reduced alveolar bone resorption within the *in vivo* model [42]. Herbal lozenges composed of *M. oleifera* leaf and *Cyanthillium cinereum* (Less.) H. Rob extracts showed an antioxidant activity with powerful effects in reducing gingivitis and oral inflammation in a double-blinded, randomized, controlled clinical trial of smoker volunteers [43]. Mouthwashes containing ethanolic leaf extracts of *Citrus hystrix*, *M. oleifera*, and *Azadirachta indica* have been shown to decrease the gingival index and plaque index with a reduction in both *Staphylococcus* and *Candida* species in gingivitis subjects after 14 days in comparison with chlorhexidine gluconate [44]. Thus, these new mouthwashes could reduce gingival inflammation and be a complementary treatment in microbial-induced gingivitis. A randomized clinical crossover study by Duarte et al. [45] found that commercially available *Moringa*-based dentifrice was associated with a significant reduction in the gingivitis and plaque index compared with miswak dentifrice in subjects with mild-to-moderate gingivitis. However, this study has several limitations, such as short experimental duration, small sample size (only 20 subjects), and absence of a control group.

It can be concluded that different parts of *M. oleifera* demonstrate anti-inflammatory properties with the potential to reduce proinflammatory mediators and control gingivitis. These studies were again limited by the absence of conventional controls. However, derivatives of medicinal plants such as *M. oleifera* are worthy of further exploration as alternative anti-inflammatory agents in the management of a range of mucosal, periodontal, and possibly pulpal inflammatory conditions.

## 5. Effect of *Moringa* Extracts in Enamel Remineralization

Only one related study was found, which investigated the remineralization effect of lyophilized extract from *M. oleifera* leaves incorporated within plain dental varnish on *in vitro*-induced enamel lesions [46]. This study found that the formulated varnish reinforced mineralization of the lesions with complete reestablishment of the enamel surface in comparison to plain and fluoride varnishes. This could be due to the high mineral contents (Ca and P) and large range of amino acids within the *M. oleifera* extract which may play a role in peptide-guided remineralization of enamel surface lesions.

## 6. Regeneration and Healing Potential of *Moringa oleifera*

Extracts from *M. oleifera* have been investigated for their capacity to induce bony growth and enhance the integration of intraosseous implants. These studies are based on the assertion that extracts of this plant leaf can act as osteoconductive and osteoinductive agents when combined with demineralized freeze-dried bovine bone xenograft. This formula was evaluated in a *Cavia cobaya* animal model which revealed superior bone regeneration and socket preservation after tooth extraction associated with significant expression of transforming growth factor-beta 1 (TGF-*β*1) and osteocalcin [47]. Extracts of acemannan and *M. oleifera*, in the form of hydrophilic gel, were used to coat titanium dental implants before implanting within the tibia and femur of rabbits. These coats were able to produce hydrophilic implant surfaces which improved bone to implant contact, stimulated new bone formation, and reduced inflammation, fibrosis, and degenerative and necrotic changes within the newly formed bone [48]. This bioactive behavior of the *M. oleifera* extract inducing bone regeneration still needs further investigations in medically compromised models such as diabetes mellitus, postradiation therapy, and osteoporosis where new bone formation may be hindered by the systemic condition of patients.

Another study was conducted to identify the healing effect of the *M. oleifera* leaf extract in gel form with two concentrations (2% and 4%) on oral excisional wounds in the palate of Sprague-Dawley rats. Povidone iodine gel (10%) was used as a control [49]. Histopathological sections from *Moringa*-treated samples revealed a faster rate of fibroblast and collagen fiber deposition in the course of the initial phase of palatal wound healing, whereas a slower rate was seen in the control group. This suggested that the *M. oleifera* leaf extract could accelerate wound healing in the oral cavity by virtue of its antibacterial and anti-inflammatory capacities. However, actual mechanisms of such actions are currently unclear and require further clarification. Also, the optimal concentration and physical stability of the formulation to be used need to be explored before its clinical application.

An isothiocyanate extracted from *M. oleifera* seeds named moringin was reported in an *in vitro* study to stimulate human periodontal ligament stem cells (hPDLSCs) into neural progenitor, osteogenic, and adipogenic activation, with no sign of tumorgenic activation [50]. Another study explored gene deregulation in the mitophagy of hPDLSCs pretreated with moringin. Most of the genes showed significant downregulation in comparison to untreated cells. Accordingly, this advantageous effect seemed to improve hPDLSCs for potential application in stem cell therapies, specifically for disorders that involve oxidative stress as pathological process mechanisms, such as neurodegenerative diseases [51].

Aqueous extracts of *M. oleifera* were found capable of enhancing proliferation, viability, and migration of human dermal fibroblast cells *in vitro* [16]. *M. oleifera* aqueous extract also enhanced wound healing under sustained hyperglycemic conditions in an animal model owing to decreased wound size, improved wound contraction and tissue regeneration, downregulation of inflammatory mediators, and upregulation of vascular endothelial growth factor [52].

Aqueous extracts of *M. oleifera* flower showed a noticeable ability in promoting rat fibroblast proliferation, bone marrow-derived mesenchymal stem cell differentiation, and angiogenesis, with no observed effect on abnormal cancer cell formation, whereas aqueous leaf extract showed lesser ability to stimulate cell proliferation but had prominent cytotoxicity on Mcf7 breast cancer cell lines [53]. In another study, the latter extract was demonstrated to decrease skin wound areas in diabetic rats and increase macrophage and fibroblast population, leading to a high level of wound contraction in comparison to a control group treated with normal saline [54]. n-Hexane extract and hydrogels of *M. oleifera* seeds showed wound healing capacity and accelerated regeneration of tissue with decreased inflammatory cells and increased vascularity of the newly formed skin in excision and incision wound-healing mouse models [55]. Topical contact of the wound surface with a gel layer of *M. oleifera* leaf extract-loaded chitosan microparticles formed a protective coat above the wound with sustained release of *M. oleifera* active constituents. This was reported to stimulate keratinocyte growth in an ex vivo pig skin model, which suggested the potential of this combination in exuding wound treatment [56]. Dressing containing 0.5% *M. oleifera* leaf extract-loaded film showed satisfactory drug release properties to facilitate the healing process of wounds in streptozotocin-induced diabetic type II rat models [57]. Furthermore, *M. oleifera* gum polysaccharide carbopol-based hydrogel dressing formulated by [58] was able to maintain a moist environment over the wound bed to enhance re-epithelialization and wound healing.

Alginate-PEG methyl ether methacrylate-*Moringa oleifera*-aloe vera scaffolds showed notable increases in the number of viable human skin fibroblast cells, compared to control scaffolds used in cell-free and cell-based assays [59].

A phenolic glycoside in *M. oleifera* seeds named 1-O-(4-hydroxymethylphenyl)-*α*-L-rhamnopyranoside MPG was tested for its hepatoprotection against carbon tetrachloride CCl4-induced hepatotoxicity in L02 cell lines (immortalized human hepatocyte cell line L02) and mice. Both *in vitro* and *in vivo* studies showed that MPG can notably protect the liver against acute hepatotoxicity induced by CCl4 through its antioxidant ability, regulating inflammatory mediators and antiapoptosis [60].

*M. oleifera* part extracts were used as a stabilizing and reducing/oxidizing agent in the biosynthesis of metal and metal oxide nanoparticles [61], with the benefit of this plant's phytochemicals, such as tannins, flavonoids, saponins, and alkaloids along with vitamins [62]. Some of the metals and metal oxides used in green synthesis of nanoparticles utilizing *M. oleifera* extracts are listed with their publications in Table 1.

Green gold nanoparticle synthesized using methanolic leaf extracts of *M. oleifera* as stabilizing agents was proved to be biologically active, less cytotoxic against blood cells *in vitro*, and helps in regeneration of neuronal cells in a Swiss albino mouse model based on the observation of their effect on brain architecture [75]. According to the authors, biosynthesis of these nanoparticles was achieved using gold chloride HAuCl_4_.3H_2_O, with the addition of methanolic extract of *M. oleifera* leaf as the stabilizing agent. The mixture was kept under continuous stirring at 75°C and then filtered and centrifuged to remove unwanted heavy materials [75]. In another study, *M. oleifera* seed polysaccharide was used to produce nanoparticles. The extract was mixed with AgNO_3_ solution and distilled water, and then, the mixture was centrifuged to completely remove unbounded compounds. A pellet was obtained that was dried in an oven at 80°C to produce nanoparticles. These nanoparticles exhibited no cytotoxic effect on mouse fibroblast L929 cells and promoted their migration *in vitro*, with antibacterial effects against wound infection pathogens. Furthermore, these nanoparticles facilitated infected wound healing with excellent biocompatibility, eliminating pathogenic bacteria and reducing inflammation and maximum epithelization after 9 days of administration in an *in vivo* male rat wound-healing model [76].

There is increasing evidence to demonstrate that *M. oleifera* extracts from different parts of the plant have the ability to promote the proliferation of various normal stem cells and other cells [77], promote wound healing and tissue regeneration, and even promote angiogenesis. These effects, combined with their antimicrobial properties, suggest that extracts of *M. oleifera* may be worthy of further investigation as therapeutic agents in the regeneration of the dentin-pulp complex after dental injury.

## 7. Conclusion

The present review summarized published information on the potential of *Moringa oleifera* extracts to control infections and support tissue healing in a range of *in vitro* and *in vivo* models. At this stage, the evidence is limited as many published laboratory studies do not exactly reflect clinical conditions. Diverse mechanisms have been suggested for these biological actions, including antioxidant, neuroprotective, anti-inflammatory, and antimicrobial activities. These may in turn have the potential to be induced by a range of biologically active derivatives including polyphenols, flavonoids, glycosides, glucosinolates, and many others. Although direct evidence is still limited, the potential of such plant derivatives in supporting wound and tissue regeneration after dental injury is worthy of further investigation.

## Figures and Tables

**Table 1:** Green synthesis of nanoparticles from *M. oleifera* extracts.

Nanoparticles	*M. oleifera* extracts used	Biological effect	References
Silver	(i) Aqueous leaf extract	(i) Broad-spectrum antimicrobial agent	[63]
	(ii) Aqueous flower extracts	(ii) Antimicrobial against *Klebsiella pneumoniae* and *Staphylococcus aureus*	[64]
	(iii) Stem bark extract	(iii) Anticancer activity against HeLa cell types	[65]

Gold	(i) Leaf extract	(i) Antioxidant, antidiabetic, and anticancer activities	[66]
	(ii) Aqueous leaf extract	(ii) Anticancer agent	[67]

Copper oxide	(i) Leaf extract	(i) Antibacterial activity against *Bacillus cereus*, *Staphylococcus aureus*, *Escherichia coli*, and *Klebsiella pneumoniae* strains	[68]
	(ii) Leaf extract	(ii) Antioxidant and cytotoxicity against cancer cells	[69]

Iron oxide	(i) Aqueous leaf extract	(i) Antibacterial agent against *Escherichia coli*, *Pseudomonas aeruginosa*, *Staphylococcus aureus*, *Shigella*, *Salmonella typhi*, and *Pasteurella multocida*	[70]
	(ii) Aqueous leaf and flower extracts	(ii) Antioxidant and antimicrobial action	[71]

Magnesium oxide	(i) Aqueous leaf extract	(i) Antibacterial and antifungal activities	[72]

Nickel oxide	(i) Aqueous leaf extract	(ii) Antibacterial and cytotoxic activities against human cancer cells	[73]

Zinc oxide	(i) Aqueous leaf extract	(i) Antibacterial against *Bacillus subtilis* and *Escherichia coli*	[74]
